# Community-directed delivery of doxycycline for the treatment of onchocerciasis in areas of co-endemicity with loiasis in Cameroon

**DOI:** 10.1186/1756-3305-2-39

**Published:** 2009-08-27

**Authors:** Samuel Wanji, Nicholas Tendongfor, Theolbald Nji, Mathias Esum, Julious N Che, Armand Nkwescheu, Fifen Alassa, Geremy Kamnang, Peter A Enyong, Mark J Taylor, Achim Hoerauf, David W Taylor

**Affiliations:** 1 Department of Biochemistry and Microbiology, University of Buea, P.O. Box 63, Buea Cameroon; 2 Research Foundation for Tropical Diseases and Environment, P.O. Box 474, Buea, Cameroon; 3 Ministry of Public Health, Division of Operational Research, Yaoundé, Cameroon; 4 Melong Health District Services, P.O. Box 68 Melong, Cameroon; 5 Mbanga Health District Services, P.O. Box 29 Mbanga, Cameroon; 6 Tropical Medicine Research Station, P.O. Box 55, Kumba Cameroon; 7 Filariasis Research Laboratory, Molecular and Biochemical Parasitology, Liverpool School of Tropical Medicine, Pembroke Place, Liverpool, L3 5QA, UK; 8 Institute of Medical Microbiology, Immunology and Parasitology (IMMIP), University Hospital Bonn, Sigmund-Freud Str.25, 53105 Bonn, Germany; 9 Centre for Infectious Diseases, College of Medicine and Veterinary Medicine, University of Edinburgh, Summerhall, Edinburgh EH9 1QH, UK

## Abstract

**Background:**

Severe side effects following ivermectin treatment of onchocerciasis in areas of co-endemicity with loaisis have been an impediment for the work of the African Programme for Onchocerciasis Control (APOC) in forested regions of several countries. Doxycycline has been shown to be effective in the treatment of onchocerciasis and has the added advantages of killing adult *Onchocerca volvulus *but neither adult *Loa loa *nor their microfilariae. This drug therefore offers great potential for the treatment of onchocerciasis in areas of co-endemicity with loiasis. The limitation of use of this drug is the duration of treatment that may pose a potential problem with therapeutic coverage and compliance with treatment. To benefit from the advantages that doxycycline offers in the treatment of onchocerciasis, it will be necessary to establish an effective distribution system that can access remote communities. This study assessed the feasibility of a large-scale distribution of doxycycline for the treatment of onchocerciasis in areas of co-endemicity with loiasis using a community-directed approach.

**Methods:**

The study was carried out in 5 health areas co-endemic for *Onchocerca volvulus *and *Loa loa *which had no prior experience of the Community Directed Treatment with Ivermectin (CDTI). The community-directed delivery process was introduced using a cascade mechanism from the central health system that passed through the regional health delegation, health district and the health areas. Community health implementers (CHIs) were trained to deliver doxycycline to community members and, under the supervision of the health system, to monitor and document drug intake and side effects.

**Results:**

The community members adhered massively to the process. Of the 21355 individuals counted, 17519 were eligible for treatment and 12936 were treated with doxycycline; giving a therapeutic coverage of eligible population of 73.8%. Of the 12936 who started the treatment, 97.5% complied by the end of six weeks. No serious side effect was registered during the six week treatment.

**Conclusion:**

This study indicates that when empowered the community health implementers can successfully deliver doxycycline for six weeks for the treatment of onchocerciasis in areas of co-endemicity with loiasis. The therapeutic coverage and the compliance treatment rate achieved in this study coupled to the known efficacy of doxycycline on *O. volvulus*, are indicators that the strategy involving the mass administration of doxycycline can be used to control onchocerciasis in those areas of co-endemicity with loiasis where ivermectin may be contraindicated.

## Background

Onchocerciasis or river blindness is endemic in many sub-Saharan African countries, with minor foci in Central and South America and the Yemen [1]. It is estimated to affect over 37 million people [2], of whom 500,000 have been visually impaired and 270,000 blind.

The registration of ivermectin as a drug for the treatment of onchocerciasis in 1987 raised great hopes for onchocerciasis control in Africa. After successful clinical trials, the pharmaceutical firm, Merck and Co, undertook to provide the drug free of charge as long as it was needed for the treatment of onchocerciasis. A new strategy involving a large-scale mass distribution of ivermectin to endemic communities [3] was rapidly put in place by APOC with an objective to eradicate onchocerciasis as a public health problem, and a barrier to socio-economic development through a community-directed treatment approach. Using this approach, over 60 million people were receiving regular treatment for onchocerciasis by the end of 2008, close to one million disability-adjusted life years have been averted, the prevalence of infection has reduced by about 73% compared with pre-APOC levels. [4].

However, during mass treatment of onchocerciasis with ivermectin in forested zones of Central Africa regions (mainly in Cameroon and the Democratic Republic of Congo), several cases of adverse reactions (encephalitis, neurologic disorders, coma and death) post-ivermectin treatment were reported in patients infected with *L. loa *[5,6]. These adverse reactions appear to be associated with high intensity of infection with *L loa *[7] and are a constraint on the implementation of CDTI in areas endemic for *O. volvulus *and *L. loa*.

The development and validation of the rapid assessment procedure for loiasis (RAPLOA) to delineate communities at risk of severe adverse reactions to ivermectin treatment was a step towards solving this problem [8]. In addition, guidelines have been developed by the Mectizan^® ^Expert Committee (MEC) and the APOC Technical Consultative Committee (TCC) and recommend special monitoring of treatment in areas where the RAPLOA prevalence is above 40% [9]. This notwithstanding, due to the remoteness of some communities in areas of co-endemicity, some cases of severe adverse effects are never detected or treated on time and have sometimes resulted in deaths. Furthermore, ivermectin is only microfilaricidal and there is growing concern that the low success of mass treatment in some areas may, in part, be due to existence of *O. volvulus *worms that show a sub- optimal response to ivermectin [10,11]

These observations have prompted a search for new filaricides which could substitute ivermectin in areas of co-endemicity and/or could be used to treat onchocerciasis without affecting *L. loa*. In this regard, the discovery of *Wolbachia *endobacteria in all pathogenic human filarial nematodes [12,13] but not *L. loa *[14,15] presented an opportunity to take a novel approach to onchocerciasis treatment based on use of antibiotics to target the endobacteria [[16,17], and [18]]. Such strategies have now been shown to be effective in both the long-term reduction of parasite loads and morbidity [18]. In animal studies, it was observed that depletion of the *Wolbachia *using tetracycline not only had microfilaricidal effects but also led to the sterility of female worms [16]. Similarly in humans, a 6-week course of doxycycline treatment against *O. volvulus *depleted the bacteria, resulted in a blockage of embryogenesis and killed the adult worms. These effects were reflected in sustained reductions in skin microfilariae [19-21]. These findings have demonstrated the superior pharmacological efficacy of doxycycline for onchocerciasis. [22] Macrofilaricides have a higher potential for sustainable onchocerciasis control but this can be only guaranteed through high coverage and compliance [23]. This is particularly important for a drug such as doxycyline that must be taken on a daily basis for 6 weeks. The argument for mass treatment with doxycycline in areas of co-endemicity with *O. volvulus/L. loa *relies heavily on its selective toxicity for *O volvulus *and the absence of adverse reactions due to lack of action on *L. loa*. However, its acceptance as a complementary tool for mass treatment of onchocerciasis will very much depend on development of effective strategies for delivery to target communities.

In a recent multi-country study carried out in five African countries, it has been demonstratated that the community-directed approach can be used to deliver ivermectin and other more complex health interventions in an integrated manner [24]. This study was designed to assess the feasibility of a large-scale delivery of doxycycline using the community-directed approach for the treatment of onchocerciasis in areas of co-endemicity with loiasis, with the specific objectives being to determine the therapeutic coverage, the compliance rate and to document the side effects associated with the treatment.

## Methods

### Study design

The study was carried out with communities in the Mbanga and Melong health districts in the littoral region of Cameroon from November 2006 to October 2008. These communities have never received mass treatment with ivermectin. A cascade mechanism involving advocacy meetings with all stakeholders (health system and community members) was used to introduce the community directed delivery process to the population in onchocerciasis/loiasis co-endemic areas. Community health implementers selected by community members were trained to deliver doxycycline to the community members and to document the treatment coverage, compliance and side effects. This work was carried out under the supervision of the health system. Treatments took place in June 2007 and in July 2008 for those who missed treatment in 2007.

### Study sites

#### Melong Health District

This health district covers a land area of 641 km^2 ^with an estimated population of 122,925 inhabitants and comprises 10 health areas. The health areas selected for the study included Mbokambo and Essekou. The health area of Mbokambo covers an area of 98 km^2 ^with an estimated population of 4128 inhabitants while Essekou covers an approximate area of 75 km^2 ^with an estimated population of 3244 inhabitants. Mbokambo and Essekou are situated in a forest zone.

#### Mbanga Health District

This District covers an area of 540 km^2 ^with an estimated population of 58,244 inhabitants and comprises 8 health areas. The health areas selected for the study included Matouke, Kotto and Mombo. These health areas are situated along the Mungo River which represents a potential breeding site for *Simulium*. The health area of Matouke is mainly occupied by the Cameroon Development Corporation (CDC) workers and has about 7183 inhabitants, Kotto 2542 inhabitants and Mombo 4258 inhabitants.

In both districts the main occupation is farming and the major cash crops are rubber, cocoa, coffee and oil palm, while food crops include cassava, groundnuts, plantains, maize and beans. The autochthonous ethnic group is Mbo but many other tribal groups (Bamileke, Esimbi, Ngi, Batibo) are present.

### Selection of study communities

A rapid epidemiological survey was conducted in the study sites to confirm the endemicity of *L. loa *and *O. volvulus*. The combined RAPLOA and Rapid epidemiological assessment of onchocerciasis (REA) protocol was used to assess the prevalence of loiasis and onchocerciasis [25]. The results obtained from this survey guided the selection of three health areas from Mbanga health district (Matouke, Kotto and Mombo) and two from Melong health district (Essekou and Mbokambo).

### Introduction of the community directed intervention

Advocacy meetings on the community-directed treatment with doxycycline were held with the health system at the central, regional, district levels and front-line health facilities. These meetings brought together representatives of the Ministry of Health, National Onchocerciasis Task Force (NOTF), Non-governmental organisations (NGO) involved in the control of onchocerciasis and the health personnel. During these meetings the project objectives were presented to the different partners and its philosophy. The role of each partner in the process was discussed and their adherence to the process was sought. Discussions also focused on the disease onchocerciasis, its socio-economic impact and its control in Cameroon.

At the community levels, social mobilisation meetings were organised in each community selected for the study to sensitise the community leaders and their subjects on the community-directed treatment process. Participants in these meetings were the community leaders and their populations, personnel of the district health service, the staff of the front line health facilities (FLHF) and social scientists. Discussions were done principally in *Pidgin English *and when necessary, translations were made into local languages.

### Training of Community Health Implementers

Community Health Implementers were selected by the community members and were trained to dispense doxycycline to community members. CHIs were also trained to: i, carry out census exercises; ii, to determine eligibility of patients for the treatment; iii, to determine treatment dosage; iv, complete treatment registers; and, v, identify and report of side effects. Training sessions were organized in the front-line health facilities. The training was done by the staff of the district health service and the medical personnel of the FLHF under the supervision of the research team.

A population census followed training of CHIs. Census was done in each community by the community health implementer. In each household all individuals were recorded. Information on age, sex, occupation and time spent in the community was collected from each individual. For adult females additional information on pregnancy or breastfeeding status was collected. This exercise was monitored by the medical personnel and the research team.

### Delivery of intervention

Doxycycline tablets (Vibramycin^®^) were from the Pfizer Company through the German medical aid organization e.v Action Medeor. The treatment regimen was 100 mg tablet per day for 42 days. Eligibility criteria for treatment included: individuals aged 12 years and above; resident in the community; not suffering from any chronic disease (hypertension, tuberculosis, diabetes, AIDS); not pregnant and not breastfeeding women. Doxycycline is not prescribed to children less than 9 years of age because it interferes with the growth of teeth. The choice of 12 years as an age limit was recommended by the institutional review board (IRB) as a measure to minimize a potential risk for participants. To ease the work of the community health implementers, the drugs were pre-packaged at the community pharmacies of the front- line health facility in appropriate daily and weekly individual doses before the commencement of treatment.

The treatment strategy adopted was door-to-door. Each week the CHI collected the week's dose from the FLHF staff and proceeded with the treatment making sure that each participant ate something before swallowing the tablet. Each treatment was recorded in a treatment register.

### Ethical and administrative clearance

This study was approved by the Ministry of Public Health of Cameroon who issued an administrative clearance (N° D30-70/L/MSP/SG/DRO/CRC). The ethical clearance was obtained from the institutional review board of the Tropical Medicine Research Station, Kumba, Cameroon. Communities and individuals who participated in the study gave their written informed consent.

### Monitoring and evaluation of treatment

The monitoring of drug intake by the patients was done on a daily basis by the CHIs and on a weekly basis by the staff of the FLHF and the research team. The staff of the FLHF and the District medical officer addressed issues of side effects reported by CHIs. At the end of the exercise, all treatment registers were retrieved for the compilation of data. Treatment records and supervision reports were used to evaluate the treatment coverage and population compliance with treatment [26,27].

Social scientists carried out in-depth interviews with health personnel, community leaders and community health implementers, while focus-group discussions were conducted with community members to assess their attitudes towards the treatment process.

### Data processing

Treatment data were entered in a template developed in Epi Info 6 and analyzed in SPSS Version 15. The analysis consisted in determining the therapeutic coverage and compliance with doxycycline treatment. Two types of therapeutic coverage were determined:

- the general therapeutic coverage was defined as the proportion of individuals treated in the total population censured;

- the therapeutic coverage of the eligible population was defined as the proportion of individuals that were treated in the eligible population.

Compliance was defined as the proportion of individuals who completed the six weeks' treatment without missing a single treatment.

The side effects observed during treatment and the frequency of occurrence of each were recorded.

## Results

### Endemicity of onchocerciasis and loiasis in the study site

The five health areas selected for the study were endemic for onchocerciasis and loiasis. Onchocerciasis was meso- or hyper- endemic in all the health areas with prevalence varying from 22% in Mbokambo to 49.7% in Matouke. The prevalence of loiasis varied from 17.9% in Matouke to 48.9% in Mombo (Table 1).

**Table 1:** Endemicity of *L. loa *and *O. volvulus *in the study areas

		**RAPLOA (Loiasis)**		**REA (Onchocerciasis)**	
		
**Health District**	**Health Area**	**No. examined**	**Prevalence (%)**	**No. examined**	**Prevalence (%)**
**Mbanga**	Kotto	282	32.1	90	48.36
	
	Matouke	480	17.93	185	49.72
	
	Mombo	466	48.95	159	41.5

**Melong**	Essekou	234	54.13	112	22.73
	
	Mbokambo	449	40.39	223	22.04

### Introduction of the Community-directed intervention (CDI) process

Advocacy meetings at national, regional, health district and FLHF levels were very important to explain the philosophy of CDI and to seek commitment of stakeholders. The Ministry of Health issued an administrative clearance and instructed the Division of Operational Research at the Ministry of Public Health to monitor the study in the field. The National Onchocerciasis Control Programme (NOCP) demonstrated its commitment and agreement to the process by exempting the study sites from its own CDTI activities and by participating in advocacy and restitution meetings. The Health System graciously integrated into their agenda the calendar of research activities. The Health System took part in all activities from advocacy meetings to the training of CHIs, pre-packaging of drugs, social mobilization, and introduction of the intervention in the communities and monitoring of the treatment.

### Training of community health implementers and population census

Community health implementers were trained and rotated during the second phase of the study. A total of 96 CHIs were trained in the health district of Mbanga and 94 in the health district of Melong. Table 2 gives the number of CHI trained in different health areas.

**Table 2:** Community Health Implementers trained and recycled in different health areas

**Health district**	**Health areas**	**Number of study communities**	**Number of CHI trained in first phase**	**Number of CHI recycled or trained in the second phase**
**Mbanga**	Kotto	3	15	11
	
	Matouke	9	48	45
	
	Mombo	6	35	24

**Melong**	Essekou	4	35	27
	
	Mbokambo	13	57	59

**Total**		**35**	**190**	**166**

Table 3 gives the population structure following a general census conducted. 21355 individuals were censured. In all, 17519 individuals were eligible for treatment. Of the 3836 individuals who were not eligible for treatment, 3354 (15.7%) were below 12 years, 435 (2.0%) were pregnant or breastfeeding women and 47 (0.22%) were individuals with chronic diseases (Table 3).

**Table 3:** Population structure after the census.

		**Non-eligible for treatment**				
			
**Health district**	**Health areas**	**Population censused**	**Children below 12 years**	**Pregnant** **/breast feeding** **women**	Individuals with chronic diseases*	**Eligible for treatment**
**Mbanga**	Kotto	2542	415	53	6	2068
	
	Matouke	7183	800	123	13	6247
	
	Mombo	4258	462	71	1	3724

**Melong**	Essekou	3244	636	94	24	2490
	
	Mbokambo	4128	1041	94	3	2990

**Total**		**21355**	**3354**	**435**	**47**	**17519**

* Chronic diseases included: Tuberculosis, AIDS, Hypertension, Diabetes or individuals on long-term antibiotics therapy.

### Therapeutic coverage and compliance with treatment

Of the 21355 individuals censused, 17519 were eligible for the treatment of which 12936 were treated with doxycycline, giving a general therapeutic coverage of 60.6% and a therapeutic coverage per eligible population of 73.83%. Table 4 gives the eligible population and the therapeutic coverage achieved during the two phases.

**Table 4:** Treatment coverage of the general and eligible population in different health areas

**Health district**	**Health areas**	**Population censused**	**Eligible population**	**Eligible population treated**	**General treatment coverage**	**Treatment overage of eligible population**
**Mbanga**	Kotto	2542	2068	1768	69.55	85.49
	
	Matouke	7183	6247	4737	65.95	75.83
	
	Mombo	4258	3724	2339	54.93	62.81

**Melong**	Essekou	3244	2490	1885	58.11	75.70
	
	Mbokambo	4128	2990	2207	53.46	73.81

**Total**		**21355**	**17519**	**12936**	**60.58**	**73.84**

Of the 12936 individuals that started treatment in the study area, 12612 completed the 6 weeks treatment without missing a single treatment, giving a compliance rate of 97.5%. The compliance rates varied from 95.46% in Kotto to 99.52% in Matouke (Table 5).

**Table 5:** Compliance rate of the population with the 6 weeks treatment with doxycycline in the different health areas.

**Health district**	**Health areas**	**Week 1**	**Week 2**	**Week 3**	**Week 4**	**Week 5**	**Week 6**	**Overall**
**Mbanga**	Kotto	99.77	96.98	95.37	94.48	93.49	92.67	**95.46**
	
	Matouke	99.96	99.69	99.58	99.42	99.29	99.21	**99.52**
	
	Mombo	100.00	99.51	99.19	98.70	98.21	97.95	**98.93**

**Melong**	Essekou	99.85	97.64	96.25	94.94	94.01	93.81	**96.11**
	
	Mbokambo	99.89	99.00	97.89	96.64	96.03	95.62	**97.49**

	**Total**	**99.89**	**98.56**	**97.66**	**96.83**	**96.20**	**95.85**	**97.50**

560 individuals dropped out of treatment (462 in the first phase and 98 in the second phase). 92 individuals who dropped out of the treatment in the first phase took part and completed the treatment in the second phase. Reasons for dropping out of the treatment after beginning of treatment were: pregnancy (1.9%); travel out of the village (18.75%); abandoned with no reason advanced (69.3%) and sickness (10.0%).

There was no significant difference in the compliance rate with respect to the age and sex of individuals.

### Side effects following doxycycline treatment

Of the 371 cases of side effects recorded during the treatment, 270 (72.8%) cases were side effects that are well known and associated with doxycycline intake including: nausea (137.4%); mild diarrhoea (12.6%); vomiting (13.7%); loss of appetite (1.1%); mild skin rash (5.9%); joint pains (7.4%); fever (4.1%); headache (6.2%); blurred vision (2.2%); and, feeling tired (9.2%).

101 (27.2%) cases reported: body pains (22.8%); boils; increase appetite (7.9%); feeling sleepy (9.9%); and, body itches (59.4%). Apart from one patient who received anti-inflammatory drugs to treat a swollen arm, these side effects were generally mild and subsided without any intervention or interruption of treatment.

## Discussion

The occurrence of severe adverse reactions in loiasis patients following ivermectin treatment [5,6] has raised major concerns for Community Directed Treatment with Ivermectin programmes situated in areas of co-endemicity. As a consequence, it has become necessary to identify new filaricides, and one approach has been to find a drug that can kill *O. volvulus *but not *L. loa*. There is sufficient scientific evidence proving the efficacy of doxycycline in the treatment of onchocerciasis [16,17,19,21,22] and critically, this drug is both micro- and macro-filaricidal for *O. volvulus *and has no effect on *L. loa *[14].

Unlike ivermectin, which requires just a single dose, doxycyline requires a course of treatment lasting 5 to 6 weeks and this presents logistical problems [28]. The main challenge remained its delivery to remote communities given its long treatment regimen. This study, which is not a clinical trial, was designed to test the feasibility of delivering doxycycline to communities using a community directed approach, a method that has previously proved effective and sustainable in the delivery of ivermectin, impregnated bed nets, and antimalarial drugs [3,24].

### Therapeutic coverage and implications for the mass administration of doxycycline for the control of onchocerciasis

In this study, using the CDI approach to deliver doxycycline, we achieved a general therapeutic coverage of 60.6% and a therapeutic coverage per eligible population of 73.8%. A high therapeutic coverage is a prerequisite for success of any disease control programme. The therapeutic coverage achieved in this study is high and augurs well for encouraging for a subsequent implementation of a large-scale distribution of doxycycline for the treatment of onchocerciasis in areas co-endemic with *L. loa*. With a yearly coverage of 65% the African programme for onchocerciasis control recommends 12 to 15 years of treatment with ivermectin (ivermectin is only microfilaricidal) to eradicate onchocerciasis [29]. Doxycycline combines the properties of being both a macro and microfilaricide on *O. volvulus *with 60 to 65% of the female worms dying following a six week treatment [30] (Turner DJ, Tendongfor N, Esum M, Johnston KL, Langley RS, Ford L, Faragher B, Walker B, Whitehead J, Specht S, Mand S, Hoerauf A, Enyong P, Wanji S, Taylor MJ: **Macrofilaricidal activity after doxycycline treatment of *Onchocerca volvulus *in people co-infected with *Loa loa*: a randomized, double-blind, placebo controlled trial**, *PLOS NTD, Submitted*). It acts on embryogenesis in female worms leading to complete sterility before the death of female worms [16]. Doxycycline retards the development of *O. volvulus *larvae in the vector as microfilariae depleted from Wolbachia endosymbiotic bacteria develop poorly in *Simulium *sp (Anna Albers, Mathias Eyong Esum, Tendongfor N, Enyong P, Wanji S, Hoerauf A, Pfarr K: **Retarded *Onchocerca volvulus *L1 to L3 larval development in the *Simulium damnosum *vector after anti-wolbachial treatment of the human host**. *Int. Journal of Parasitology*, *Submitted*). Mathematical simulations show that macrofilaricidal drugs have higher potential of achieving elimination of onchocerciasis and require less number of rounds of mass drug administration [31]. It can therefore be anticipated that with a general therapeutic coverage of 60% achieved with the community-directed administration of doxycycline it may require less round of mass drug administration to bring the community microfilarial load of *O. volvulus *below the threshold level of transmission in a given endemic area. Nevertheless, it is necessary to develop a mathematical model based on data generated so far to determine the number of rounds as well as the periodicity of mass administration of doxycycline for the control of onchocerciasis.

### High treatment compliance rate and lack of severe side effects as indicators of sustainability for the large-scale use of doxycycline through the community-directed approach

Complying or conforming to treatments is absolutely necessary for effective medical treatment to have its desired effects. Good compliance to treatment is dependent on the willingness and motivation of patients to follow the regimens prescribed, which is based on the information about the medication received by the patient, the complexity of the regimen, the side effects and the benefits they may derive from the treatment [32-34]. In this study 97.5% of the people who started treatment effectively completed it. This compliance was high despite the long regimen of doxycycline and also is a good indicator that doxycycline can be mass administered for the control of onchocerciasis using a community directed approach. The compliance rate did not vary with age or sex of the treated population. This is an indication of good acceptability of the drug by all categories of individuals in the community. Community members reported that doxycycline relieves them from body itches, nodules, improved their vision, increased their appetite and sexual desire, cured boils and wounds. The positive feed-back from community members who took part in the first phase of treatment motivated reluctant individuals to adhere to treatment in the second phase. Some individuals who took the treatment in the first phase were very willing to repeat in the second phase as a result of the benefits derived "*many people are asking for the drugs in my community and those who have already been treated are still requesting for another treatment" *said one CHI in Sanke. Doxycycline being a broad spectrum antibiotic, in addition to its filaricidal effect, had extended effects on some common bacterial infections. Pregnancy, travel, illness and fear of side effects were the main reasons for refusing or interrupting the treatment.

Several factors accounted for the high compliance rate achieved in this study. The social awareness campaigns in which the population was well informed on the process and the role to play in the process contributed much to its success. The fact that each partner in the CDI process adequately played his or her role was an important factor for success. The awareness of the burden of the disease onchocerciasis and its socio-economic impacts on the population was a motivating factor for community adherence and compliance to treatment "*before the advent of doxycycline distribution, filarial was more serious than any other disease in this community*" declared a community member. The fact that the drug was delivered by CHIs selected by community members guaranteed some trust and was a great motivating factor in the population's adherence and compliance with the treatment. It is known that interventions delivered by the community health workers are more accepted by the population than those delivered using the normal health system [35] and that it significantly improves the accessibility of the treatment and treatment seeking behaviour of community members [36,37].

The free distribution of the drug was also an encouraging factor for the population to accept and adhere to the treatment. The positive effects experienced by those who took the drug motivated others to request for their own treatment, "*I used to have a nodule on my knee, but it has disappeared after doxycycline treatment"*, declared a notable of the Mbokambo community who took the treatment 24 months ago.

Doxycycline today offers a good alternative to ivermectin in the treatment of onchocerciasis in areas of co-endemicity with loiasis. In fact during the six weeks mass treatment with doxycycline, the side effects recorded were generally well known side effects associated with doxycycline intake (nausea, diarrhoea, vomiting, skin rash blurred vision, and headache). These side effects were mild and subsided without any medical intervention or interruption of the treatment. This study has largely demonstrated that the intake of doxycycline at a large scale is accompanied by mild side effects and this can justify the use of this drug to treat onchocerciasis in areas where *O. volvulus *and *L. loa *co-exist. The high compliance rate recorded in this study and the lacks of severe side effects are good indicators that the mass administration of doxycycline for the treatment of onchocerciasis in areas of co-endemicity of loiasis can be a sustainable activity.

### Potential advantage in cost-effectiveness of the community-directed approach

In order for any health intervention to achieve its goal, the intervention must be cost-effective. That is affordable to the health system, donors or community members. Policy and decision makers are increasingly requesting information on cost-effectiveness to minimise cost of interventions and to efficiently allocate health resources. Few studies have investigated the cost effectiveness of community- based interventions. A recent multi-country study on community-directed interventions for major health problems in Africa involving eight research teams from five African countries demonstrated that community-directed interventions is more cost-effective than conventional delivery systems for the integrated delivery of vitamin A, insecticide treated nets and antimalarial drugs [24]. With lower implementation costs, the community directed approach achieved higher coverage than the conventional delivery system at the health district and front line health facility levels. Other more recent studies confirmed those findings with community based interventions producing economic savings and health benefits that are sustained over long period [37-39]. Though our study was not designed to measure the cost of large- scale delivery of doxycycline, it is very likely that the implementation of the delivery of doxycycline using the community-directed approach will be more cost- effective than if it were to be done with the conventional delivery system at the health district and front-line health facility levels. One important consideration for the large scale use of doxycycline for the treatment of onchocerciasis is the fact that this drug is not presently made available free of charge as compared to ivermectin. The cost (purchase and delivery) of the six week treatment per patient is estimated at $ 2.5 (data not shown). Given the fact that most of the onchocerciasis patients living in endemic communities have income below the poverty level; this estimated cost may be a serious barrier to their accessibility to this therapy. It would therefore be necessary for the health systems of endemic countries to subsidise the cost of doxycycline for the end- users in the large scale treatment of onchocerciasis where appropriate.

## Conclusions and perspectives

This study indicates that, when empowered, the community health implementers can successfully deliver doxycycline for six weeks for the treatment of onchocerciasis in areas of co-endemicity with loiasis. The therapeutic coverage and the compliance treatment rate achieved in this study coupled to the known efficacy of doxycycline on *O. volvulus*, are indicators that the strategy involving the mass administration of doxycycline can be used to control onchocerciasis in those areas of co-endemicity with loiasis where ivermectin may be contraindicated. Such community-directed approach is likely to be more cost-effective than the one achieved through conventional health system. It is necessary to develop a mathematical model based on data generated from this study coupled to other studies on efficacy of doxycycline on *O. volvulus *both in the human host and *Simulium *vector to define the number of rounds and the periodicity of mass administration of doxycycline to control onchocerciasis.

## Abbreviations

APOC: African Programme for Onchocerciasis Control; CDC: Cameroon Development Corporation; CDI: Community Directed Interventions; CDTI: Community Directed Treatment with Ivermectin; CHI: Community Health Implementer; FLHF: Front Line Health Facility; IRB: Institutional Review Board; MEC: Mectizan^® ^Expert Committee; NGO: Non Governmental Organization; NOTF: National Onchocerciasis Task force; NOCP: National Onchocerciasis Control Programme; RAPLOA: Rapid Assessment Procedure of Loiasis; REA: Rapid Epidemiological Assessment of Onchocerciasis; TCC: Technical Consultative Committee.

## Competing interests

The authors declare that they have no competing interests.

## Authors' contributions

SW designed the study, coordinated the field activities, interpreted the results and edited the manuscript. NT participated in the study design, collection of data, and analysis of data and drafted the manuscript. TN participated in social mobilization, data collection, and interpretation of social science data. ME participated in evaluation of the endemicity of onchocerciasis and loiasis in study communities, drafting of manuscript. CJN participated in data collection in the field. AN participated in clinical examination of patients before treatment. FA supervised field activities (delivery of drugs to health centres, pre-packaging of drugs and delivery to communities and monitoring of side effects). JK supervised field activities (delivery of drugs to health centres, pre-packaging of drugs and delivery to communities and monitoring of side effects). PE participated in the study design and co-edited the manuscript. TMJ edited the manuscript. AH participated in the study design and edited the manuscript. TWD participated in the study design, ordered doxycycline from Pfizer, carried out field visits and edited the manuscript.

## References

[B1] World Health Organization (1995). Onchocerciasis and its control. WHO Tech Rep Ser.

[B2] Basanez MG, Pion SD, Churcher TS, Breitling LP, Little MP, Boussinesq M (2006). River blindness: a success story under threat?. PLoS Med.

[B3] World health Organization, Geneva (1996). Community-directed treatment with ivermectin: report of a multi-country study. Special Programme for Research and Training in Tropical Diseases.

[B4] http://www.who.int/apoc/cdti/achievements/en/index.html.

[B5] Chippaux JP, Boussinesq M, Gardon J, Gardon-Wendel N, Ernould JC (1996). Severe adverse reaction risks during mass treatment with ivermectin in loiasis-endemic areas. Parasitology Today.

[B6] Gardon J, Gardon-Wendel M, Demanga ND, Kamgno J, Chippaux JP, Boussinesq M (1997). Serious reactions after mass treatment of onchocerciasis with ivermectin in an area endemic for *Loa loa *infection. Lancet.

[B7] Boussinesq M, Gardon J, Kamgno J, Pion SDS, Gardon-Wendel N, Chippaux JP (2001). Relationship between the prevalence and intensity of *Loa loa *infection in the Central Province of Cameroon. Annals of Tropical Medicine and Parasitology.

[B8] Wanji S, Takougang I, Yenshu EV, Meremikwu M, Enyong P, Braide E (2001). Rapid Assessment Procedures for Loiasis. Report of a Multi-centre study. UNDP/World Bank/WHO Special Programme for Research and Training in Tropical Diseases TDR/IDE/RP/RAPL/011.

[B9] MEC/TCC Guidelines (2004). Recommendations for the treatment of Onchocerciasis with Mectizan^® ^in areas co-endemic for Onchocerciasis and Loiasis. http://www.mectizan.org/loarecs.asp.

[B10] Awadzi K, Boakye DA, Edwards G, Opoku NO, Attah SK, Osei-Atweneboana MY (2004). An investigation of persistent microfilaridermias despite multiple treatments with ivermectin, in two onchocerciasis-endemic foci in Ghana. Ann Trop Med Parasitol.

[B11] Awadzi K, Attah SK, Addy ET, Opoku NO, Quartey BT, Lazdins-Helds JK (2004). Thirty-month follow-up of suboptimal responders to multiple treatments with ivermectin, in two onchocerciasis-endemic foci in Ghana. Ann Trop Med Parasitol.

[B12] Taylor MJ, Hoerauf A (1999). Wolbachia bacteria of filarial nematodes. Parasitol Today.

[B13] Taylor MJ, Bandi C, Hoerauf A (2005). Wolbachia bacterial endosymbionts of filarial nematodes. Adv Parasitol.

[B14] Büttner DW, Wanji S, Bazzocchi C, Bain O, Fischer P (2003). Obligatory symbiotic Wolbachia endobacteria are absent from *Loa loa*. Filaria J.

[B15] McGarry HF, Pfarr K, Egerton G, Hoerauf A, Akue JP, Enyong P Evidence against Wolbachia symbiosis in *Loa loa*. Filaria J.

[B16] Hoerauf A, Volkmann L, Hamelmann C, Adjei O, Autenrieth IB, Fleischer B (2000). Endosymbiotic bacteria in worms as targets for a novel chemotherapy in filariasis. Lancet.

[B17] Langworthy S, Renz A, Mackentedt U, Henkle-Duhrsen K, de Bronsvoort- MB, Tanya VN (2000). Macrofilaricidal activity of tetracycline against the filarial nematode *Onchocerca ochengi*: elimination of Wolbachia precedes worm death and suggests a dependent relationship. Proc Roy Soc London.

[B18] Hoerauf A, Mand S, Volkmann L, Buttner M, Marfo-Debrekyei Y, Taylor M (2003). Doxycycline in the treatment of human onchocerciasis: Kinetics of Wolbachia endobacteria reduction and inhibition of embryogenesis in female Onchocerca worms. Microbes Infect.

[B19] Hoerauf A, Mand S, Adjei O, Fleischer B, Büttner DW (2001). Depletion of Wolbachia endobacteria in *Onchocerca volvulus *by doxycycline and microfilaridermia after ivermectin treatment. Lancet.

[B20] Debrah AY, Mand S, Marfo-Debrekyei YM, Larbi J, Adjei O, Hoerauf A (2006). Assessment of microfilarial loads in the skin of onchocerciasis patients after treatment with different regimens of doxycycline plus ivermectin. Filaria J.

[B21] Turner JD, Mand S, Debrah AY, Muehlfeld J, Pfarr K, McGarry HF (2006). A randomized, double-blind clinical trial of a 3-week course of doxycycline plus albendazole and ivermectin for the treatment of *Wuchereria bancrofti *infection. Clinical Infectious Diseases.

[B22] Hoerauf A (2006). New strategies to combat filariasis. Expert Rev Anti Infect Ther.

[B23] Alley WS, van Oortmarssen GJ, Boatin BA, Nagelkerke NJ, Plaisier AP, Remme JH (2001). Macrofilaricides and onchocerciasis control: Mathematical modelling of the prospects for elimination. BMC Public health.

[B24] World Health Organization Special Programme for Research and Training in Tropical Diseases (2008). Community-directed interventions for major health problems in Africa. A multi-country study, Final Report.

[B25] Wanji S, Tendongfor N, Esum M, Yundze SS, Taylor MJ, Enyong P (2005). Combined Utilization of Rapid Assessment Procedures for Loiasis (RAPLOA) and Onchocerciasis (REA) in Rain forest Villages of Cameroon. Filarial J.

[B26] Brieger WR, Okeibunor JC, Abiose AO, Ndyomugyenyi R, Kisoka W, Wanji S, Elhassan E, Amazigo UV (2007). Feasibility of measuring compliance to annual ivermectin treatment in the African Programme for Onchocerciasis Control. Tropical Medicine and International Health.

[B27] Amazigo UV, Obono OM, Dadzie KY, Remme JHF, Jiya J, Ndyomugyenyi R (2002). Monitoring community-directed treatment Programmes for sustainability: Lessons from the African Programme for Onchocerciasis Control (APOC). Annals of Tropical Medicine and Parasitology.

[B28] Hoerauf A, Specht S, Marfo-Debrekyei Y, Büttner M, Debrah AY, Mand S (2008). Efficacy of 5-week doxycycline treatment on adult *Onchocerca volvulus*. Parasitol Res.

[B29] Awadzi K, Attah SK, Addy ET, Opoku NO, Quartey BT (1999). The effects of high-dose ivermectin regimens on *Onchocerca volvulus *in onchocerciasis patients. Trans R Soc Trop Med Hyg.

[B30] Hoerauf A, Specht S, Marfo-Debrekyei Y, Büttner M, Debrah AY, Mand S, Batsa L, Brattig N, Konadu P, Bandi C, Fimmers R, Adjei O, Büttner DW (2008). Efficacy of 5-week doxycycline treatment on adult Onchocerca volvulus. Parasitol Res.

[B31] Alley WS, van Oortmarssen GJ, Boatin BA, Nagelkerke NJ, Plaisier AP, Remme JH, Lazdins J, Borsboom GJ, Habbema JD (2001). Macrofilaricides and onchocerciasis control, mathematical modeling of the prospects for elimination. BMC Public Health.

[B32] Depoortere E, Guthmann JP, Presse J (2005). Efficacy and effectiveness of the combination of sulfadoxine/pyrimethamine and a 3-day course of artesunate for the treatment of uncomplicated *falciparum *malaria in a refugee settlement in Zambia. Tropical Medicine and International Health.

[B33] Weiden PJ, Rao N (2005). Teaching medication compliance to psychiatric residents: placing an orphan topic into a training curriculum. Academic Psychiatry.

[B34] Yuang Y, L'italien G, Mukherjee J, Iloeje U (2006). Determinants of discontinuation of initial highly active antiretroviral therapy regimen in a US patient cohort. HIV medicine.

[B35] Gyapong MJO, Gyapong OG (2000). Community-directed treatment: the way forward to eliminating lymphatic filariasis as public-health problem in Ghana. Annals of Tropical Medicine & Parasitology.

[B36] Khalid AE, Elfatih MM, Tarig A, Salah HA, Abdalla HE, Mahmoud AM (2009). A Feasibility and acceptability of home-based management of malaria strategy adapted to Sudan's condition using artemisin-based combination therapy and rapid diagnostic test. Malaria Journal.

[B37] Amazigo U, Okeibunor J, Matovu V, Zoure H, Bump J, Seketeli A (2007). Performance of predictors: Evaluating sustainability in community-directed treatment projects of the African programme for onchocerciasis control. Social Science & Medicine.

[B38] Ashworth A (2006). Efficacy and effectiveness of community-based treatment of severe malnutrition. Food Nutr Bull.

[B39] Bachmann MO (2009). Cost effectiveness of community-based therapeutic care for children with severe acute malnutrition in Zambia: decision tree model. Cost Eff Resour Alloc.

